# *Cuminum cyminum* as green corrosion inhibitor for API 5 L X70 carbon steel in 0.5 M H_2_SO_4_ solution

**DOI:** 10.1038/s41598-025-98407-z

**Published:** 2025-05-17

**Authors:** A. S. Fouda, S. Rashwan, H. Ibrahim, M. Reda, M. E. Eissa, A. El-Hossiany

**Affiliations:** 1 Chemistry Department, Faculty of Science, Mansoura University, Mansoura, 35516 Egypt; 2 Department of Chemistry, Faculty of Science, Suez Canal University, Ismailia, Egypt; 3 Chemistry Department, College of Science, Imam Mohammad Ibn Saud Islamic University (IMSIU), Riyadh, 11623 Saudi Arabia; 4 Delta for Fertilizers and Chemical Industries, Talkha, Dakahleya Egypt

**Keywords:** Corrosion inhibition, Carbon steel, H_2_SO_4_, *Cuminum cyminum* extract, Langmuir isotherm, Adsorption, Corrosion, Materials science

## Abstract

*Cuminum cyminum* extract (CCE) has been studied as a corrosion inhibitor in controlling corrosion of C-steel corrosion in 0.5 M H_2_SO_4_, by weight loss (WL), potentiodynamic polarization (PDP), electrochemical impedance spectroscopy (EIS), energy-dispersive X-ray spectroscopy, scanning electron microscopy (SEM), and atomic force microscopy (AFM) measurements. The findings demonstrated that inhibition efficiency (% IE) enhanced with increasing extract dosages, attaining 83.49% at 200 ppm and finishing at 91.83% at 400 ppm. However, inhibition efficiency was reduced by raising the temperature, decreasing from 91.83% at 298 K to 78.63% at 318 K (all at 400 ppm). CCE adhered to the C-steel surface by the Langmuir adsorption isotherm. EIS measurements exhibited increased transfer resistance (R_ct_) as the extract dose increased. Polarization curves showed that CCE functions as a mixed-type inhibitor. Several thermodynamic parameters were computed and examined. The free energy of adsorption for the extract under study was calculated and was found 34.7–35.8 kJ mol^− 1^. Chemical and electrochemical approaches yielded reliable and agreeable results.

**Supplementary Information:**

The online version contains supplementary material available at 10.1038/s41598-025-98407-z.

Owing to its economic viability, exceptional strength, and resistance to shock, carbon steel stands as one of the most frequently used engineering materials in all industrial sectors, especially within the petrochemical industry^1^. “This makes it a popular option for pipelines that transport gas and oil, boilers, storage tanks, reactors, and heat-exchanging operations^2^. Chemical reactions between carbon steel and its environment cause it to corrode. This process is influenced by oxidation, moisture, acids, impurities, and electrical stress, among other things. Thus, one of the most expensive and risky processes that endangers all sectors is corrosion. Whereas it may cause buildings and bridges to collapse, oil pipelines to burst, chemical facilities to leak, etc^3^. One way to think of corrosion is as a substance organic breakdown brought on by an unintentional interaction with its surroundings^4^. Corrosion arises from electrochemical reactions where metal surface atoms relinquish electrons to an electron acceptor (like oxygen, acids, or cations of less active metals) present in air or aqueous solutions. This oxidation process deteriorates the entire metal surface^5^. It is commonly recognized that the kind of metal or the alloy’s metallurgical composition affects the pace of corrosion^6^. Many inhibitors are composed of organic compounds containing heteroatoms like nitrogen, oxygen, and sulfur, which can form heterocyclic compounds characterized by the presence of π-electrons^7,8^. Usually corrosion inhibition has been managed with the use of several synthetic compounds. However, the environmental and health risks connected with synthetic inhibitors have resulted in a growing desire for green and sustainable alternatives^9,10^. Therefore, a major trend in materials science and chemistry is the move toward environmentally friendly and sustainable corrosion inhibitors. Corrosion inhibitors now include natural extracts, plant-based chemicals, and other environmentally acceptable substances. These substitutes not only mitigate hazards to the environment and human health, but they also frequently present economical options. Green inhibitors are becoming more and more unlikely to utilize chemicals that could be harmful to the environment. The majority of ecological inhibitors have increased significantly during the past 20 years and are biodegradable, non-toxic, and easily accessible^11,12^. Extracts derived from the leaves of Lycoris radiata and Lycoris chinensis have exhibited efficacy in inhibiting carbon steel corrosion in acidic solutions containing hydrofluoric acid (HF), hydrochloric acid (HCl), and chloride salts. This protective effect, arising from a synergistic interaction among the compounds present in the leaves, culminated in a maximum corrosion inhibition of 91.5% for steel immersed in a 5% hydrochloric acid solution at 35°C^13^. Pineapple crown extract, rich in phytochemicals and polyphenols, provided excellent corrosion protection for steel-39 in 1 M sulfuric and hydrochloric acid solutions. The extract’s inhibitory effect, attributed to the adsorption of phytochemical components, reached a maximum of 76.8% at 3 g/L after 3 hours. Increasing the extract dose further improved inhibition^14^. *Leaves from Lycoris radiata* and Lycoris chinensis have demonstrated significant potential in preventing carbon steel corrosion in acidic solutions containing hydrofluoric acid (HF), hydrochloric acid (HCl), and chloride salts. This protective effect, resulting from the combined action of various compounds present in the leaves, yielded a maximum corrosion inhibition of 91.5% for steel immersed in a 5% hydrochloric acid solution at 35°C^15^. An *extract from Calopogonium mucunoides* demonstrated significant potential as a corrosion inhibitor for mild steel in a highly corrosive 0.5 M hydrochloric acid solution. A maximum inhibition efficiency of 91.4% was achieved using an optimal extract dose of 1.2 g/L at a temperature of 25°C. However, the inhibitory effect diminished with increasing temperatures. The Langmuir isotherm model accurately described the adsorption behavior of the extract onto the steel surface^16^. Kavitha and her team reported using an *aqueous Rosa Damascena extract* to inhibit mild steel corrosion in oil-water environments. The highest level of corrosion inhibition, 96%, was achieved using a 0.1 M extract dose. Increasing the dose of the extract further enhanced its inhibitory effect. This extract exhibited mixed-type inhibition, leading to the formation of a protective barrier layer on the metal’s surface^17^. The corrosion of carbon steel in a 1 M sulfuric acid solution was investigated using an extract from *Azadiracta indica* leaves, commonly known as Neem. The leaves were extracted with ethanol and assessed for their ability to prevent corrosion. It was determined that the dose of the extract significantly influenced its corrosion inhibition effectiveness, with higher doses leading to an increase in inhibition efficiency from 41–86%^18^. *Rumex extract* strongly inhibits C38 steel corrosion in harsh 1 M HCl. Optimal conditions of 30°C and 2 g/L yield a maximum efficiency of 94.6%. The mechanism involves adsorption and electrochemical effects. Future research should identify active compounds, refine extraction, and assess long-term performance. Additionally, cucumber peel extract (CPE) and cucumber seed oil (CSO) show promise for AISI 1007 steel corrosion inhibition^19^.*The CPE and CSO* inhibitors effectively reduce C38 steel corrosion in seawater, achieving peak inhibition of 94.4% and 95.4% at 1.0 g/L dose. The mixed-type inhibition mechanism suggests both adsorption and electrochemical effects. The Langmuir and Dubinin-Radushkevich isotherms describe the adsorption behavior. However, the effectiveness decreases over time, indicating potential limitations for long-term applications. Further research is needed to optimize the inhibitor formulation and understand the degradation mechanisms^20^. *Spinacia oleracea extract*^21^ effectively inhibits carbon steel corrosion in 1.0 M HCl, reaching 93% efficiency at 500 ppm. Increasing the dose reduces ferric ion concentration. Langmuir isotherm suggests monolayer film formation. *Terminalia Arjuna*, a medicinal herb, inhibits mild steel corrosion in 0.2 M HCl^22^, reaching 64.1% efficiency at 30°C for 3 days, offering a greener alternative to synthetic inhibitors. When submerged in acidic solutions such as 0.5 M HCl and 0.5 M H_2_SO_4_, displayed significant corrosion inhibition capabilities. At a concentration of 0.75 g/L and a temperature of 30°C, it achieved an impressive inhibition efficiency of 84%. This compound functions as a mixed-type inhibitor, forming a protective film on the metal surface. However, the inhibitory effect diminishes as the concentration of reactants or inhibitors increases and as the temperature rises^23^. The methanolic extract derived from Equisetum hyemale exhibited notable corrosion resistance properties when applied to mild steel immersed in 1 M HCl. The extract’s inhibitory effectiveness initially showed an upward trend with increasing temperature, culminating in a maximum efficiency of 85% at a dose of 1000 ppm within the initial 6 hours of immersion. This level of effectiveness remained relatively stable until the 12-hour mark. The adsorption process underlying this inhibitory effect was determined to be spontaneous, endothermic, and of a mixed-type nature. The inhibitor molecules adsorbed onto the steel surface as a monolayer, adhering to the Langmuir isotherm^24^. *Date palm leaf extracts*, when extracted with various organic solvents like ethanol, acetone, and butanol, demonstrated significant corrosion inhibition properties for low-carbon steel in a highly corrosive 1 M HCl solution at 25°C. Among these solvents, butanol proved to be the most effective, yielding a maximum inhibition rate of 82% at a dose of 400 ppm. The inhibitory efficiency generally increased with increasing extract dose, reaching a remarkable 97% protection at a dose of 1000 ppm. However, the inhibition performance slightly declined at higher temperatures, dropping to 86% at both 40°C and 50°C, and further decreasing to 82% at 60°C^25^. *Paederia Foetida leaf extract* exhibits significant efficacy in inhibiting the corrosion of mild steel in 1 M HCl solutions. It achieved a maximum inhibition efficiency of 73.77% after three days of exposure, likely due to the physical adsorption of its phytochemical components onto the steel surface, forming a protective barrier against corrosion. However, prolonged exposure to the extract resulted in a gradual decline in its inhibitory performance^26^. Jaddoa et al.^27^ studied an aqueous turmeric powder extract as a corrosion inhibitor to control low carbon steel corrosion in 3.5% NaCl. They found that at 1200 ppm turmeric extract the % inhibition reachedto 97.52%. Shi et al.^28^ investgate the inhibitory properties of rapeseed cake meal extract (RCME) on the corrosion of cold rolled steel (CRS) in trichloroacetic acid (TCA) using different methods. The results demonstrate that RCME exhibits excellent inhibitory performance with a maximum inhibition efficiency of 92.7% for 100 mg L^−1^ RCME at 20°C. Wang et al.^29^ prepare fish waste extract (FWE) from acid hydrolysis with alkaline leaching using fish waste as the raw material. The results of the characterization analysis identified the presence of 17 amino acids, with Leucine, Phenylalanine, Methionine, and Alanine being the most abundant. FWE was then tested as a corrosion inhibitor (CI) for carbon steel in 0.5 mol/L H_2_SO_4_. Further, the effect of KI on the corrosion inhibition performance of FWE for carbon steel in 0.5 mol/L H_2_SO_4_ was systematically investigated. The maximum corrosion inhibition efficiencies were 88.7% and 63.9% for the FWE and KI alone, and 97.10% for the combination of FWE and KI. Döner et al.^30^ studied the Methanol extract of Arum dioscoridis (AD) as corrosion inhibitor for mild steel (MS) in 1 M HCl. Inhibition efficiency was reached the value of 97% at 1000 ppm .

*Cuminum Cyminum seeds* are employed extensively as a culinary ingredient, adding flavor to a wide range of dishes, including meats, sausages, confectionery, and bread. Additionally, they function as a natural preservative in food products. From a medicinal standpoint, cumin has been recognized for its efficacy in treating digestive disorders such as flatulence and diarrhea, as well as in wound healing^31^. *Cumin aldehyde*,* P-cymene*,* cuminal*,* α-pinene*,* and α*,* β-dihydroxyethylbenzene* are the main components found in CCE species (Fig. S1).

The primary objective of this study is to examine the influence of CCE on the corrosion inhibition properties of C-steel immersed in 0.5 M H_2_SO_4_ solution, employing WL, PDP, and EIS techniques. Furthermore, the corrosion mechanism was assessed through adsorption isotherms, activation, and thermodynamic parameters.

## Materials and procedures

### Preparation of plant extract

Cumin seeds were collected from various regions of Daqahlia Governorate in January 2023 and identified at the Botanical Herbarium at Sadat City University. The dried and ground seeds were extracted using methanol, a potent solvent for plant components^32^. 200 g of the powdered cumin seeds were soaked in 800 ml of methanol for 48 h at room temperature. The extract was then dried using a rotary evaporator under vacuum^33^. The cumin seeds were collected, dried, and ground into a fine powder. This powder was soaked in methanol and left for 48 h. The extract was then concentrated and stored in a refrigerator. The main functional groups in the extract were identified using EDX.

### Solutions

In a 500 mL measuring flask, stock solutions with an inhibitor dose of 1000 ppm were created by dissolving an equivalent amount of extract and finishing with bidistilled water. The investigated extract was diluted from stock solution and utilized at the following varied doses: 200, 250, 300, 350, and 400 ppm. An appropriate dose or H_2_SO_4_ (0.5 M H_2_SO_4_, BDF) 98% was prepared in bidistilled water. The corrosive solution was freshly made.

### C-steel specimens

C-steel API 5 L X70 (C max 0.28, Mn max 1.40, P max 0.030, S max 0.030, Ni max 0.50, Gr max 0.50, Cu max 0.50. The sum of other elements should be ≤ 0.06%, with the balance Fe) with coupons with 2 × 2 × 0.2 cm and 1 × 1 cm2 dimensions were prepared for WL and electrochemical studies.

### WL measurement

Weight loss corrosion measurements were carried out according to the ASTM procedure^34^. “A common and better technique used in corrosion laboratories to evaluate how well inhibitors reduce corrosion resistance is WL testing. After sanding the C-steel coupons with varying grit levels (P220–P2000 ISO/FEPA Grit) until a mirror surface was obvious, the coupons were rinsed with bidistilled water and allowed to air dry at room temperature. To determine the WL, metal coupons were weighed before and after immersion in 100 ml of 0.5 M sulfuric acid solutions containing various concentrations of the cumin seed extract or no extract. This process was repeated every 30 minutes for a total of 6 hours. At 298, 303, 308, 313, and 318 K, among various temperatures, the study was carried out. The following formulas were used to estimate the surface coverage (h), corrosion rate (CR), and percentage (IE%)^35.^

1 $$\:\text{C}\text{R}\:=\frac{W}{At}$$

2 $$\:{\uptheta\:}\:=\frac{CR-CR\left(i\right)}{CR}$$

3 $${\text{IE}}\% {\text{ }}={\text{ }}\theta {\text{ }} \times {\text{ 1}}00$$

“W represents the weight loss of the C-steel in mg. CR(i) and CR denote the corrosion rates (mg cm^−2^ h^−1^) in the presence and absence of the extract, respectively. A is the total surface area of the coupon in cm^2^, and t is the immersion time in hours”.

### Electrochemical measurements

To conduct electrochemical measurements, a Gamry Reference 3000 Potentiostat/ Galvanostat/.

ZRA analyzer was used. “A three-electrode cell was set up, consisting of a saturated calomel electrode (SCE) as the reference electrode, a platinum wire as the auxiliary electrode, and a C-steel API 5L X 70 electrodes as the working electrode. The working electrode was carefully prepared and secured in a glass sheath. Experiments were performed in 0.5 M sulfuric acid solutions with and without varying doses of an extract at a constant temperature of 298 K. Open circuit potential (OCP) measurements were initially conducted to attain a steady-state potential prior to commencing the electrochemical experiments. The working electrode was immersed in the test solution for duration of 30 minutes to facilitate stabilization. To further analyze the corrosion behavior, potentiodynamic polarization (PDP) and electrochemical impedance spectroscopy (EIS) techniques were employed. For PDP measurements, a potential sweep was applied, ranging from − 250 mV to + 250 mV relative to the open-circuit potential (OCP) at a scan rate of 0.2 mV/s. The corrosion current density (i_corr_) was recorded under steady-state conditions. A Tafel plot was generated to visualize the relationship between the corrosion current density and the polarization potential. The IE_PDP_ % can be calculated using the following equations^36,37^.

4 $$\:{{\%}\text{I}\text{E}}_{\text{P}\text{D}\text{P}}=\frac{{i}_{corr}-\:{i}_{corr}^{\circ}}{{i}_{corr}}\:\times\:100$$

Where i_corr_ and i^o^_corr_ are the currents in the absence and presence of CCE, correspondingly.

Electrochemical impedance spectroscopy (EIS) measurements were conducted at the open-circuit potential (OCP) using a multifrequency AC technique with a frequency range of 100 kHz to 0.2 Hz and a 10 mV amplitude signal. The EIS data provided information about the double-layer capacitance, solution resistance, and polarization resistance. The corrosion rate was estimated based on the polarization resistance^38^. The EIS results were graphically represented using Nyquist and Bode plots. The following formula^39^ was used to determine the % IE_EIS_ of CCE based on the values of (R_ct_) and (R_s_) for C-steel in a 0.5 M H_2_SO_4_ corrosive media:

5 $$\:{\text{\%}\text{I}\text{E}}_{\text{E}\text{I}\text{S}}=\frac{{\text{R}}_{\text{c}\text{t}}-{{\text{R}}^{\text{*}}}_{\text{c}\text{t}}}{{\text{R}}_{\text{c}\text{t}}}\times\:100$$

When CCE is present, the electron charge resistance is represented by R_ct (inh)_, but in the absence of CCE, R_ct_ represents the charge transfer resistance.

### Surface examination studies

#### SEM and EDX analyses

SEM-EDX analysis was employed to examine the surface morphology and elemental composition of glossy and corroded C-steel samples, both inhibited and uninhibited. The samples were immersed in 0.5 M H_2_SO_4_ for 6 h, with and without the optimal dose of the inhibitor. After drying, the samples were analyzed using SEM and EDX to study the inhibitor’s effect on the surface morphology and to confirm the elemental composition.

### Outcomes and explanation

#### WL method studies

##### Effect of inhibitor dose

**Fig. 1:**
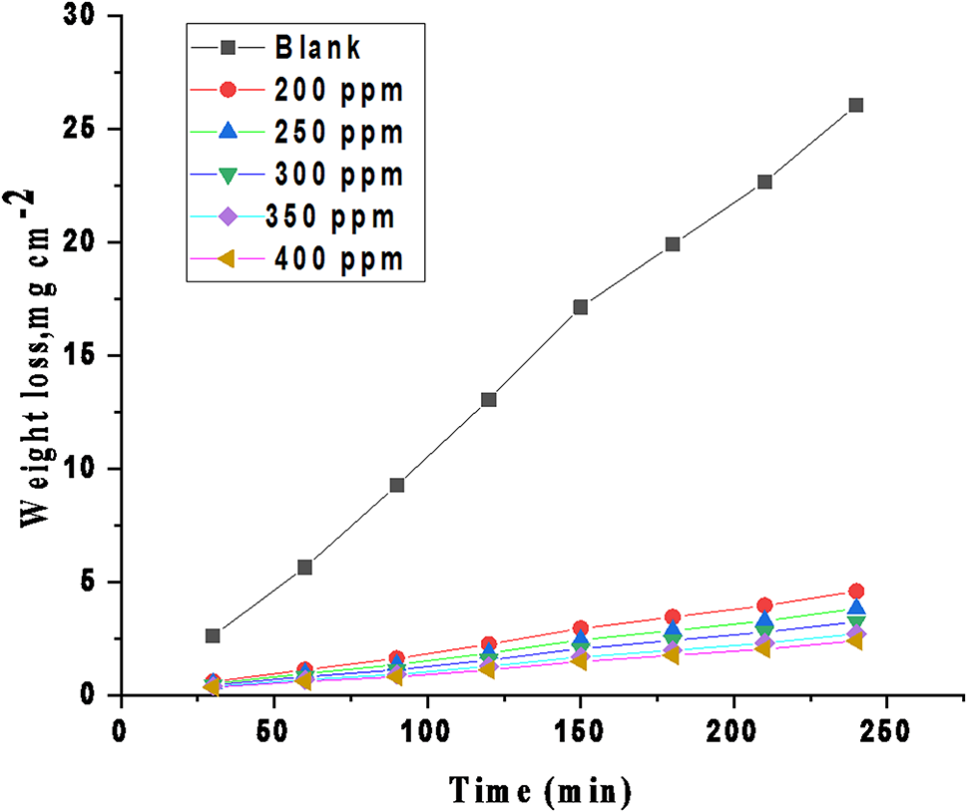
WL versus the immersion time (min) for C-steel in in 0.5 M H_2_SO_4_ at altered doses of CCE at 298 K.

Figure 1 shows the WL –time curves for various doses of CCE in 0.5 M H_2_SO_4_ solution. The protection efficiency (% IE) rises and k_corr_ falls as the dose of CCE is increased to an optimal level of 400 ppm. “Subsequent increases in extract dose do not result in substantial changes in % IE or k_corr_. The CCE extract achieved a maximum IE of 91.83% in HCl at the optimal dose of 400 ppm. This could be explained by increasing C-steel surface area coverage by the extract molecules. At the maximum dose, the corrosion rate, as measured by weight loss, is lowest, while the inhibition efficiency is highest. Further increments in extract dose did not yield significant changes in the extract’s performance. When the CCE dose exceeds 400 ppm (the optimal dose), the solution becomes saturated. There’s also the potential for a vigorous interaction between the CCE attached to the metal surface and the molecular extract in the solution^40,41^. This possibility could lead to the release of the CCE coating into the solution.

**Table 1:** Corrosion parameters determined experimentally from WL method for C-steel dissolution in 0.5 M H_2_SO_4_ solution with and without CCE at altered temperatures.

Temp. (K)	[Inhibitor] ppm	k_corr_, mg cm^− 2^ h^−1^	Ө	%IE_w_
298	Blank	6.524	–	–
	200	1.077	0.8349	83.49
	250	0.887	0.8641	86.41
	300	0.742	0.8862	88.62
	350	0.609	0.9067	90.67
	400	0.533	0.9183	91.83
303	Blank	9.570	–	–
	200	1.819	0.8099	80.99
	250	1.522	0.8410	84.10
	300	1.286	0.8656	86.56
	350	1.083	0.8868	88.68
	400	0.935	0.9023	90.23
308	Blank	14.155	–	–
	200	2.961	0.7908	79.08
	250	2.515	0.8223	82.23
	300	2.129	0.8496	84.96
	350	1.816	0.8717	87.17
	400	1.578	0.8885	88.85
313	Blank	16.035	–	–
	200	4.171	0.7399	73.99
	250	3.693	0.7697	76.97
	300	3.262	0.7966	79.66
	350	2.920	0.8179	81.79
	400	2.537	0.8418	84.18
318	Blank	20.325	–	–
	200	6.301	0.6900	69.00
	250	5.677	0.7207	72.07
	300	5.104	0.7489	74.89
	350	4.695	0.7690	76.90
	400	4.343	0.7863	78.63

##### Effect of immersion time

**Fig. 2:**
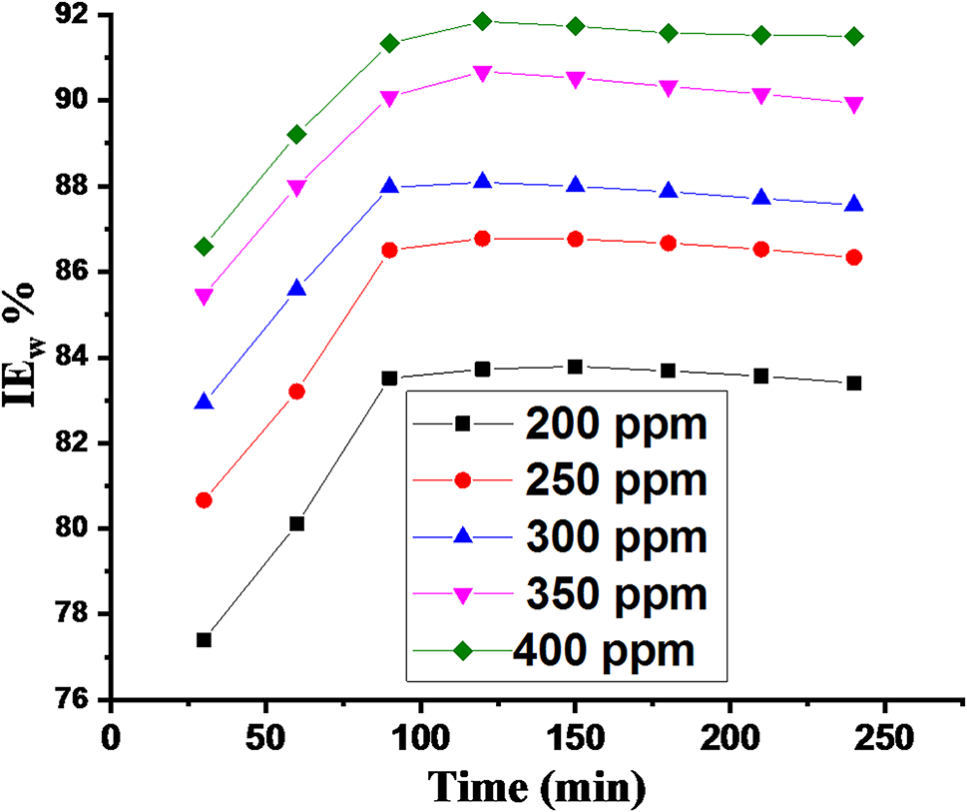
IE % vs. time of immersion at 25 °C.

To assess the long-term effectiveness of the CCE as a corrosion inhibitor, WL measurements were conducted in 0.5 M H_2_SO_4_ solution with and without the CCE over a 4-hour period at 298 K. As shown in Fig. 2, the inhibition efficiency (IE) increase with time up to 100 min and then gradually decreased with increasing immersion time. This indicates that while the CCE is effective as a short-term corrosion inhibitor for C-steel in 0.5 M H_2_SO_4_ solution, its long-term efficacy may be limited.

##### Impact of temperature

To evaluate the stability of the protective layer formed by the extract and to quantify the activation energy of the corrosion process, “weight loss measurements were carried out at temperatures ranging from 298 K to 318 K, both in the absence and presence of the optimal dose of the CCE. The immersion duration for these measurements was 4 hours. Table 1 presents the impact of temperature on the corrosion rate constant (k_corr_) of C-steel in the absence and presence of various concentrations of the extract under investigation. Based on the findings presented in this table, it is evident that an increase in temperature leads to an elevation in the corrosion rate constant (k_corr_) while simultaneously decreasing the inhibition efficiency (% IE) of the CCE. This behavior suggests that the adsorption of the plant extract onto the metal surface is primarily governed by physisorption. The relationship between the corrosion rate and temperature adheres to the Arrhenius equation, implying a linear correlation between the logarithm of the corrosion rate and the reciprocal of temperature”. This linear relationship signifies that the corrosion rate accelerates exponentially as the temperature increases^42^:

6 $$\:\text{l}\text{o}\text{g}\:\text{C}\text{R}=\left(\frac{-{\text{E}}_{\text{a}}^{\text{*}}}{2.303\text{R}\text{T}}\right)+\:\text{l}\text{o}\text{g}\:\text{A}$$

**Fig. 3:**
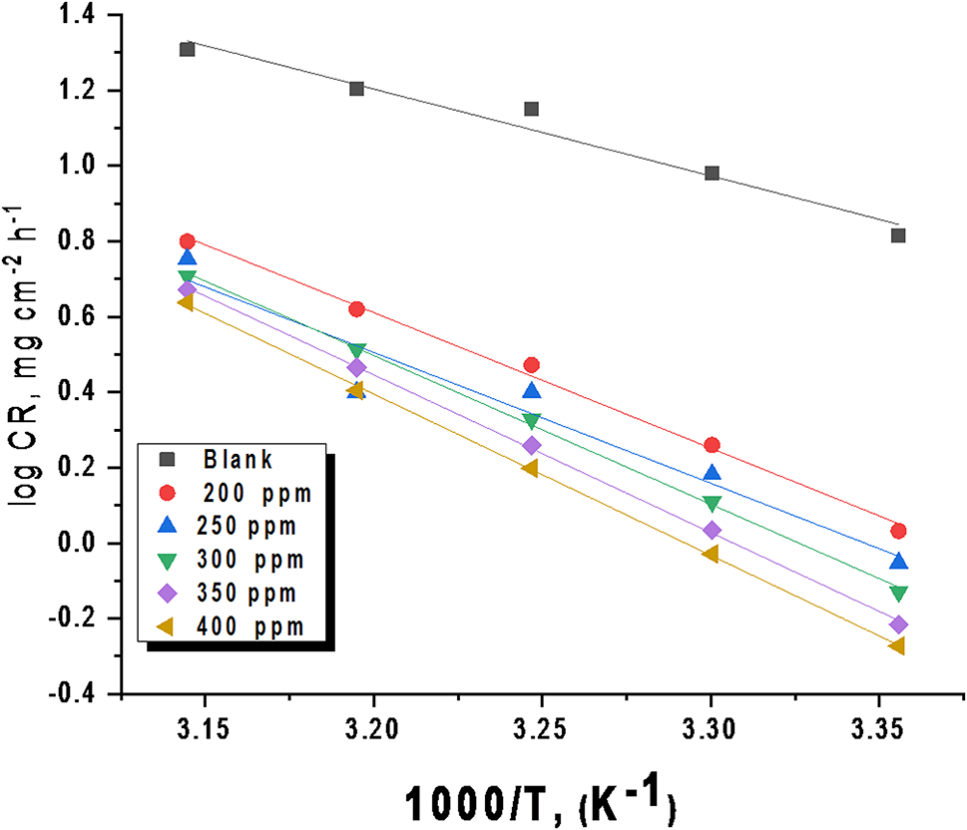
Arrhenius plots (log CR vs. 1/T) for steel dissolution in the presence an absence different doses of CCE in 0.5 M H_2_SO_4_ solution.

The given equation is the Arrhenius equation applied to corrosion kinetics. CR is the corrosion rate constant (from WL experiments), E^*^_a_ is the activation energy, T is the absolute temperature, R is the gas constant, and A is the pre-exponential factor. By measuring corrosion rates at different temperatures, you can determine E^*^_a_ and A to understand corrosion mechanisms and predict rates. Plotting the log of corrosion rate against 1/T gives a straight line (Fig. 3). The slope of this line equals –E^*^_a_/(2.303R), where E^*^_a_ is the apparent activation energy and R is the gas constant. This relationship is based on the Arrhenius equation, which relates reaction rates to temperature and activation energy. Table 2 shows the calculated activation energies. The activation energy for C-steel corrosion in 0.5 M sulfuric acid is notably higher when CCE is present. This indicates that CCE acts as a corrosion inhibitor by raising the energy barrier needed for the corrosion reaction to proceed. The higher apparent activation energy for steel dissolution in the inhibited solution suggests physical adsorption, a common initial stage of corrosion inhibition^43^. Szauer and Brand^44^ proposed that this increased energy barrier is due to reduced adsorption of the extract onto the steel surface as temperature rises. An alternative formulation of the Arrhenius equation is given by^45^:

7 $$\:\frac{\text{l}\text{o}\text{g}\text{C}\text{R}}{\text{T}}=\text{log}\left(\frac{\text{R}}{\text{N}\text{h}}\right)+\frac{\varDelta\:{\text{S}}^{\text{*}}}{2.303\text{R}}-\frac{\varDelta\:{\text{H}}^{\text{*}}}{2.303\text{R}\text{T}}$$

**Fig. 4:**
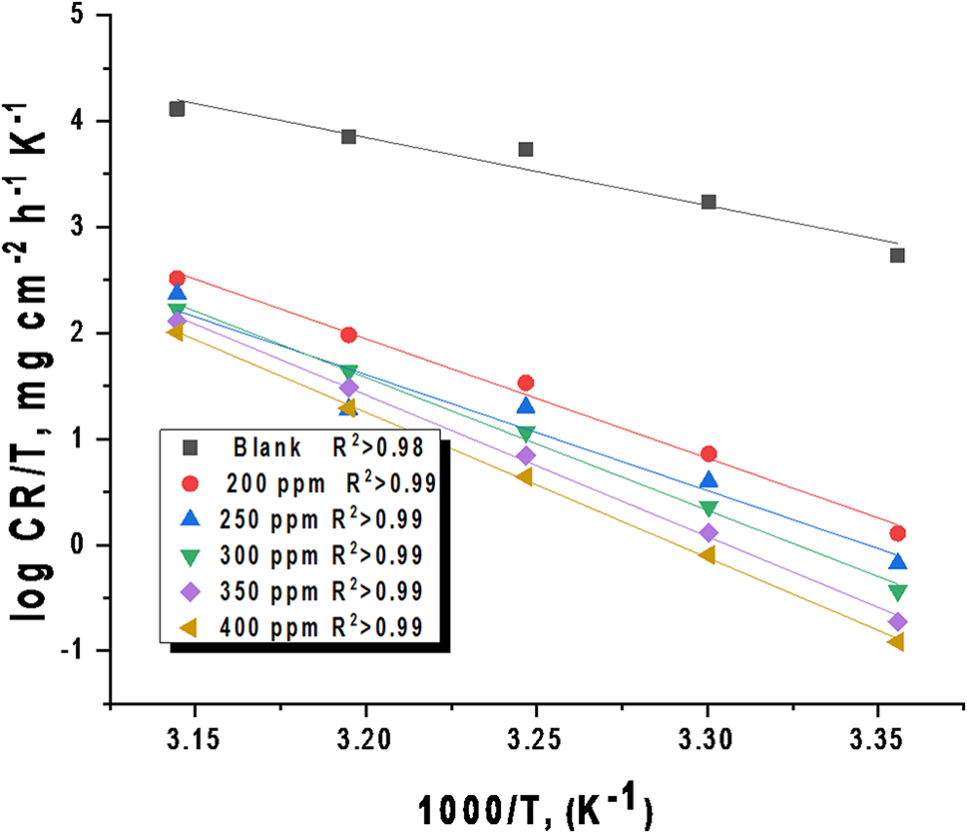
Transition state plots for C-steel dissolution in the presence and absence altered doses of CCE in 0.5 M H_2_SO_4_ solution.

In this context, h denotes Planck’s constant, with a value of 6.6260755 × 10^−34^ J s, while N represents “Avogadro’s number, valued at 6.02 × 10^23^. The symbols ∆S^*^ and ∆H^*^ represent the activated entropy and enthalpy, respectively. Figure 4 illustrates the linear relationship between the logarithm of the corrosion rate divided by temperature (log CR/T) and the reciprocal of temperature (1/T) for the extract under investigation. The values of the activated entropy (∆S^*^) and the activated enthalpy (∆H^*^) can be derived from the intercept (log (R/Nh) + ∆S*/2.303R) and the slope (−∆H^*^/2.303R), respectively. The obtained results are tabulated in Table 2. The positive values of ΔH* provide confirmation that the formation of the activated complex is an endothermic process^46^. This implies a higher energy barrier for C-steel dissolution in 0.5 M H_2_SO_4_ when CCE is present. Moreover, (∆H^*^–E^*^_a_*)* = Δn RT, R represents the gas constant, T denotes the absolute temperature, and Δn signifies the difference in the number of moles between the products and reactants. The average difference values of the (∆H^*^–E^*^_a_) parameter were determined to be 2.7 kJ mol^−1^ for C-steel immersed in 0.5 M H_2_SO_4_ in the presence of CCE. The negative shift observed in the ΔS^*^ values suggests that the formation of the activated complex involves an associative step rather than a dissociative one. This implies an increase in the system’s order as the reactants transition into the activated complex^47^. The increase in the E^*^, ΔH^*^ and ΔS^*^values by adding small doses of the CCE molecules to the corrosion medium compared to the blank solution were calculated. These indicate the formation of protective layer of CCE on C-steel surfaceby adding the CCE molecules to the corrosion mediumthus achieving more energy barrier of the corrosion reaction, compared to the blank solution, which in turn reduces the corrosion rate.

**Table 2:** Thermodynamic parameters for C-steel dissolution in a 0.5 M H_2_SO_4_ solution at altered doses of CCE measured from WL tests.

[Extract] ppm	E^*^_a_	∆H^*^	− ∆S^*^
	(kJ mol^−1^)	(kJ mol^−1^)	(J mol^−1^ K^−1^)
blank	56.06	53.36	116.93
200	96.37	93.67	230.12
250	93.66	90.96	238.63
300	106.82	104.12	260.50
350	113.79	111.09	281.46
400	116.74	114.04	289.53

##### Adsorption isotherm study

**Fig. 5:**
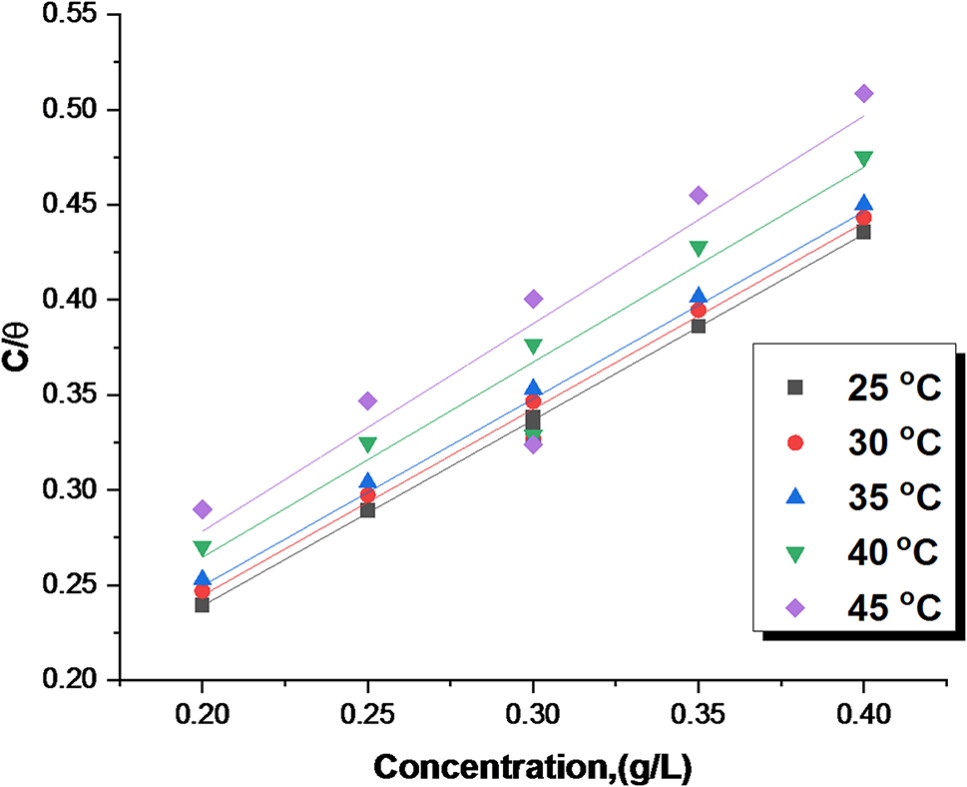
Langmuir adsorption isotherm model on the C-steel surface of CCE in 0.5 M H_2_SO_4_ at alterd temperatures.

The adsorption behavior of inhibitors on the C-steel surface can be modeled using various isotherms, such as Frumkin, Langmuir, and Temkin^48^.It’s worth noting that the Langmuir isotherm exhibits a correlation coefficient and slope that are nearly equal to unity, as illustrated in Fig. 5. This behavior can be described by the following equation (Eq. 8)^49^.

8 $$\:\frac{{C}_{inh}}{\theta\:}=\frac{1}{{K}_{ads}}+\:{C}_{inh}$$

K_ads_ is the adsorption equilibrium constant, which is derived from the intercepts of linear plots C_inh_ /θ against C_inh_ at different temperatures, as shown in Fig. 5. The elevated K_ads_ values (Table 3) point to significant adsorption of the CCE onto the C-steel surface, leading to enhanced corrosion inhibition (% IE). These values exhibit a positive correlation with increasing CCE dosages^50^. The ΔG°_ads_ values, calculated from Eq. 9, provide thermodynamic insights into the adsorption process.

9 $$\:{\text{K}}_{\text{a}\text{d}\text{s}}=\left(\frac{1}{55.5}\right)\:\text{e}\text{x}\text{p}\left(\frac{{-\varDelta\:\text{G}}_{\text{a}\text{d}\text{s}}^{\circ}}{\text{R}\text{T}}\right)\:\:$$

T and R are represent the absolute temperature and the gas constant, respectively. The concentration of the solution was maintained at 55.5 mol/L of H_2_O. The calculated values of the adsorption equilibrium constant (K_ads_) and the standard free energy of adsorption (ΔG°_ads_) are summarized in Table 3. The magnitude of ΔG°_ads_ can provide valuable insights into the adsorption mechanism. Values ranging from − 20 kJ/mol to -40 kJ/mol are typically characteristic of physisorption and chemosorption (mixed one). Less negative values below 20 kJ mol^−1^ are indicative to physisorption, which involves electrostatic interactions between charged species. More negative values, below − 40 kJ/mol, are indicative of chemisorption, involving coordination interactions between the inhibitor and the metal surface^51,52^. The calculated ΔG°_ads_ values for CCE, which fall within the range of -20 to -40 kJ/mol, suggest a mixed adsorption mechanism dominated by physisorption. The negative sign associated with these values signifies that the adsorption process of CCE is spontaneous and the adsorbed layers formed on the C-steel surface are relatively stable. The observed decrease in K_ads_ values with increasing temperature further supports this conclusion, indicating weaker adsorption at higher temperatures. Predicting adsorption behavior using natural product inhibitors is challenging based solely on the free energy of adsorption (ΔG°_ads_) values. This difficulty arises from the imprecise knowledge of the chemical composition of the adsorbed inhibitor components. However, alternative thermodynamic parameters, such as activation energy (E^*^_a_), can provide a more suitable approach for determining the adsorption mechanism of the inhibitor onto the metal surface.”

**Table 3:** Langmuir adsorption parameters for CCE at altered temperatures.

Temp., K	K_ads,_ (mL^−1^ L)	-∆Gº_ads,_ (kJ mol^−1^)
298	224	34.7
303	193	34.9
308	175	35.3
313	148	35.4
318	137	35.8

### Electrochemical studies

#### PDP studies

**Fig. 6:**
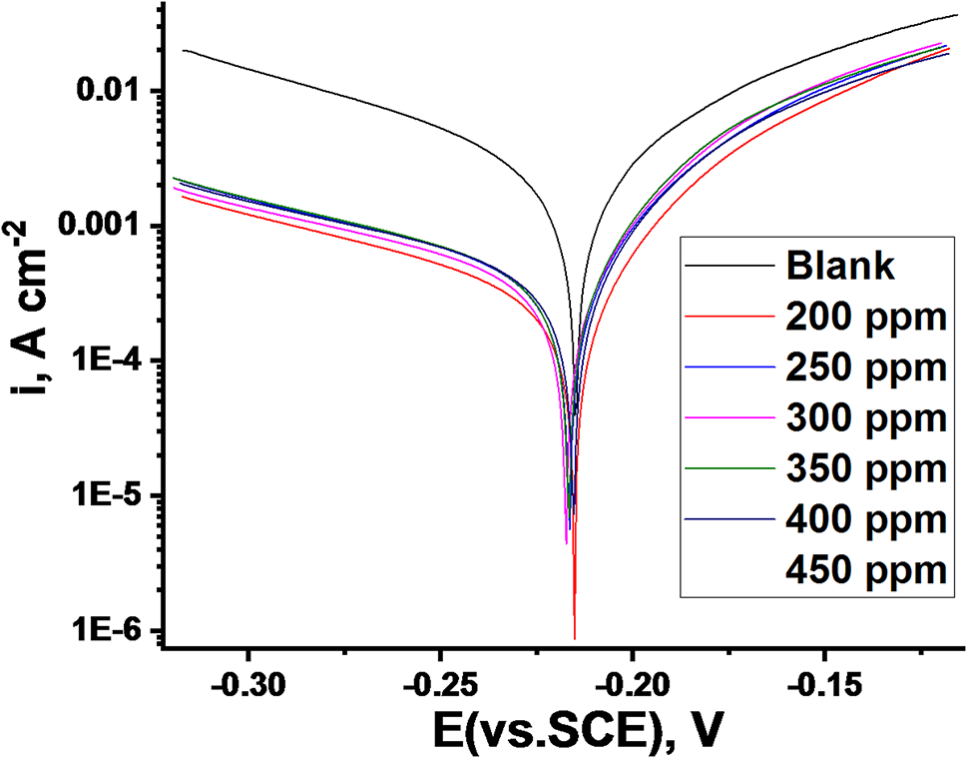
PDP plots of C-steel corrosion in acidic solution presence and absence at various doses of CCE at 298 K.

Figure 6 illustrates the PDP curves for C-steel immersed in a 0.5 M H_2_SO_4_ corrosive medium, both in the absence and presence of varying doses of CCE inhibitor at 298 K. By extrapolating the linear portions of the anodic and cathodic Tafel regions to their intersection point, we can determine the corrosion current density (i_corr_). This parameter is vital for quantifying the rate of metal dissolution and evaluating the efficacy of corrosion inhibitor. The electrochemical parameters, including anodic and cathodic Tafel slopes, were extracted from the polarization curves (Table 4). The presence of CCE led to a significant decrease in corrosion current density (i_corr_), signifying a reduction in the overall corrosion rate. This decrease, coupled with rising in IE_PDP_ %, can be attributed to the adsorption of CCE molecules onto the C-steel surface. This adsorption process forms a protective film that acts as a barrier, hindering both the anodic dissolution of the metal and the cathodic reduction of hydrogen ions. This physical barrier effectively reduces the active surface area available for electrochemical reactions, thereby mitigating corrosion. When the extract was added at a concentration of 400 ppm, the inhibitor molecules form a uniform layer on the metal surface, significantly reducing the current density. This concentration proves to be optimal for inhibition efficiency. However, increasing the concentration to 450 ppm diminishes this efficacy, which is likely attributable to the physical adsorption mechanism. At concentration exceeding 400 ppm, the solution becomes saturated with inhibitor molecules. Additionally, strong interactions between the adsorbed inhibitor molecules and those in the solution^53,54^ can potentially cause the inhibitor coating to detach from the metal surface. The corrosion potential (E_corr_) was shifted slightly towards the cathodic region, with a maximum displacement of 5 mV, suggesting that CCE acts as a mixed-type inhibitor. Changes in both anodic (β_a_) and cathodic (β_c_) Tafel slopes indicate that CCE adsorption modifies both anodic dissolution and cathodic hydrogen evolution mechanisms. Figure 6 clearly shows inhibition of both cathodic and anodic reactions, with increased inhibition at higher CCE doses. The linear polarization data is summarized in Table 4.

**Table 4:** PDP for C-steel in 0.5 M H_2_SO_4_ in the absence and presence of altered CCE at its optimum dose.

Conc., ppm	i_corr_, µA m^−2^	β_a_, mV dec^−1^	-β_c_, mV dec^−1^	-E_corr_ (mV vs. SCE)	k_corr_ mpy	θ	IE_PDP_/%
Blank	3459	49	248	220	95.5	–	–
200	495	34	193	218	7.2	0.857	85.7
250	476	37	183	219	6.9	0.862	86.2
300	426	34	171	217	6.5	0.877	87.7
350	380	34	168	216	6.2	0.890	89.0
400	311	33	160	215	4.5	0.910	91.0
450	380	35	163	217	6.2	0.890	89.0

#### EIS studies

**Fig. 7:**
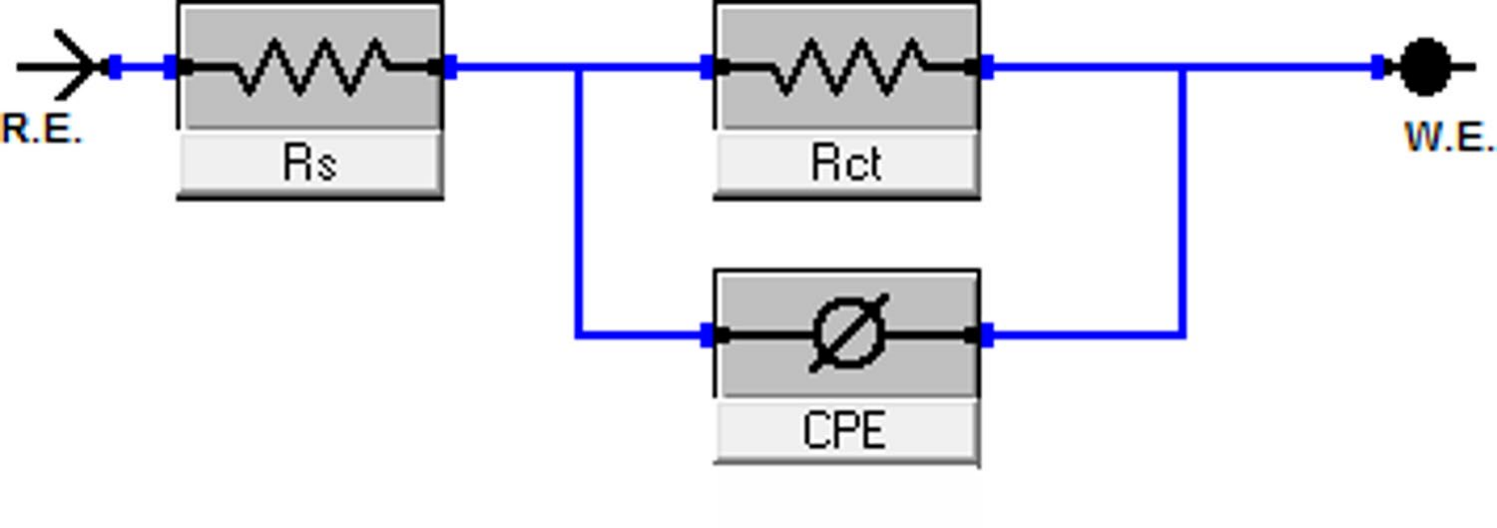
Electrochemical circuit equivalent utilized to fit EIS.

**Fig. 8:**
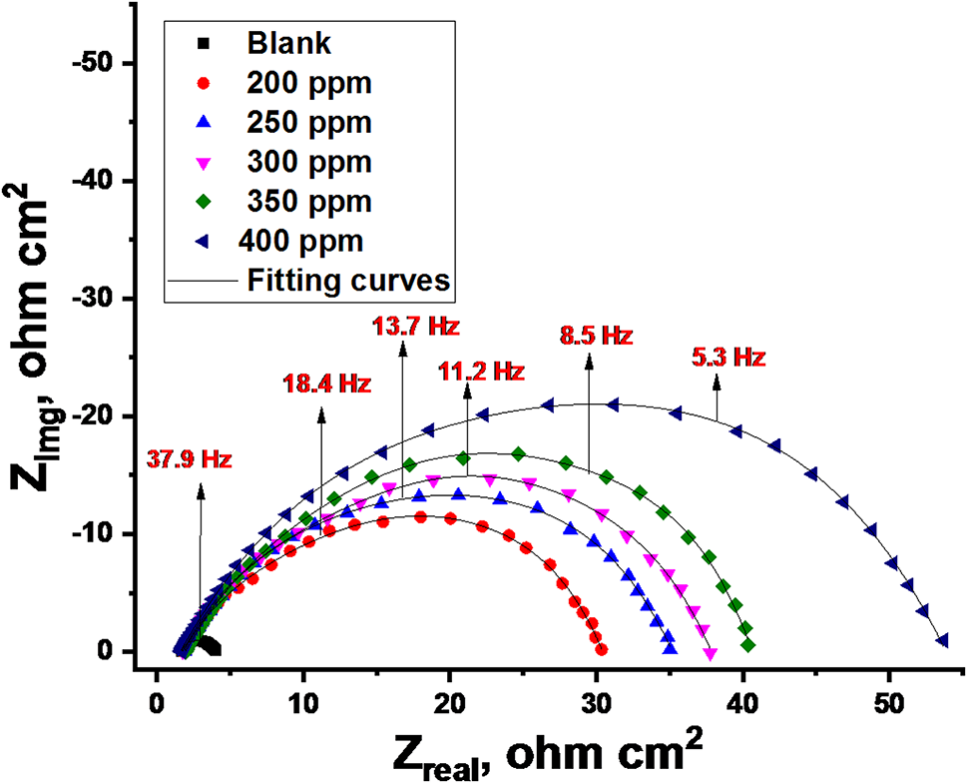
Nyquist plots for C-steel dissolution in acidic solution in the absence and presence of altered doses of CCE at 298 K.

**Fig. 9:**
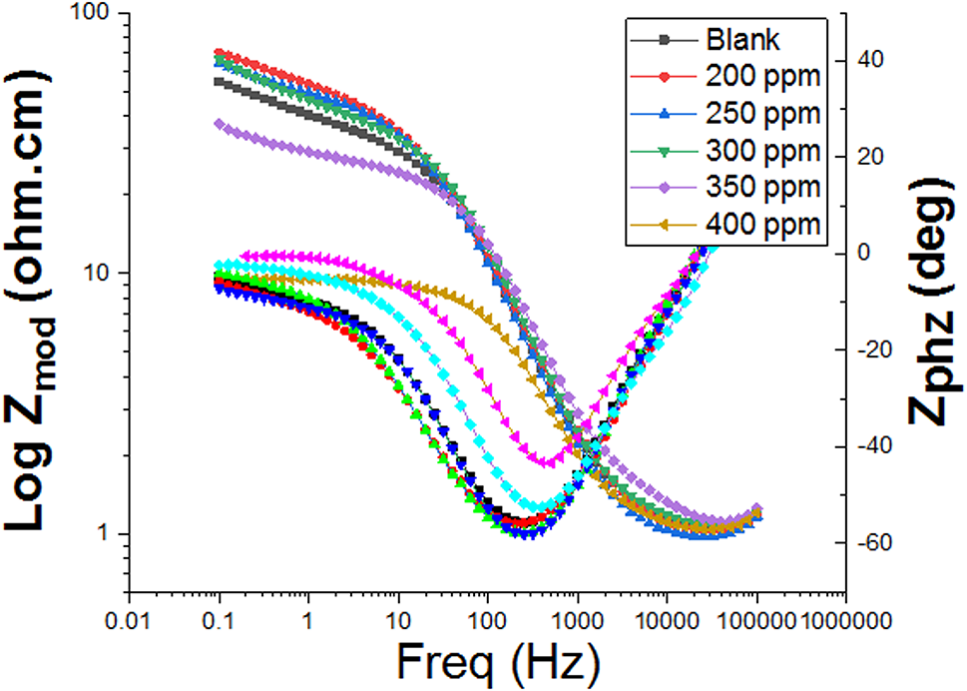
Bode plot for C-steel dissolution in acidic solution in the absence and presence of altered doses of the CCE at 298 K.

The impedance spectra were fitted to an equivalent circuit model (Fig. 7), which is commonly used to characterize the iron/acid interface, to extract valuable information about the corrosion process. The equivalent circuit model (Fig. 7) used to analyze the impedance data consists of R_s_ (solution resistance), R_ct_ (charge transfer resistance), and CPE (constant phase element). Figures 8 and 9 show Nyquist and Bode plots for the system with and without CCE. In these Figs. at various doses, the same plot was obtained, but for each dose, the intensity or height varies. The building of a protective layer from the extract on the steel surface, which lowers the corrosion rate for C-steel in the used corrosive medium, may be the cause of the higher dose giving a higher peak, which indicates stronger resistance for the employed solution. According to the Nyquist plots’ curve, the capacitive loops’ width increases with extract dose and is greater when extract is present than in its absence. Due to the frequency dispersion effect, these capacitive loops are clearly not ideal semicircles. The results in Table 5 showed that when CCE dose increases, the charge transfer resistance (R_ct_) across the metal surface increases, and the difference between (R_ct_) and (R_s_) becomes quite significant^55^. The findings of the EIS show that when the inhibitor’s dose rises, the rate of corrosion falls, and, as a result, the inhibitor’s efficacy rises.

**Table 5:** EIS parameters of C-steel corrosion acidic solution in the absence and with various doses of CCE.

Conc., ppm	Y_o_,10^-6^ µΩ^−1^ s^*n*^ cm^−2^	*n*	*R*_ct_, Ω cm^2^	C_dl_, μF cm^2^	θ	IE%	Goodness of fit (χ^2^)
Blank	500	0.974	4.5	425	–	–	16.33 × 10^−3^
200	235	0.932	30.4	164	0.852	85.2	18.39 × 10^−3^
250	226	0.924	35.1	152	0.872	87.2	20.81 × 10^−3^
300	220	0.920	37.8	145	0.881	88.1	19.75 × 10^−3^
350	205	0.916	40.3	132	0.888	88.8	17.11 × 10^−3^
400	198	0.905	53.7	122	0.916	91.6	15.77 × 10^−3^

### Surface examinations

#### SEM analysis

**Fig. 10:**
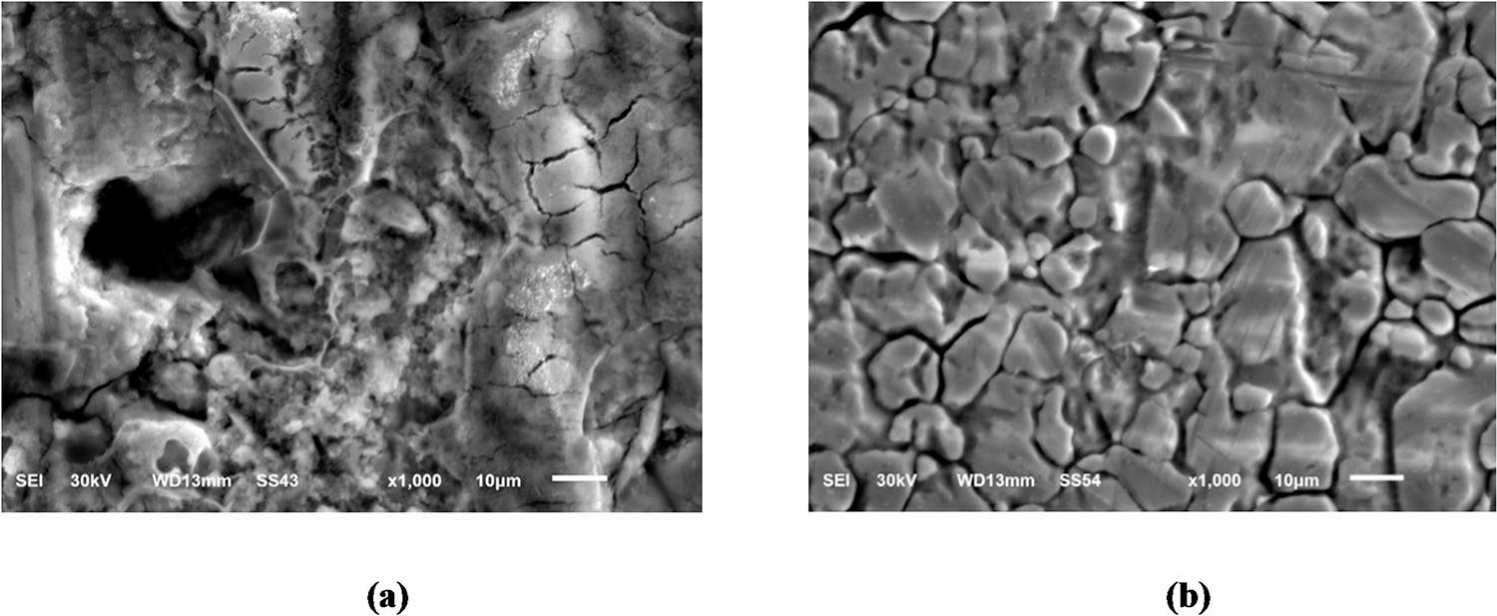
SEM profiles for: C-steel surface dipping in 0.5 M sulfuric acid solution for 24 h in the absence (**a**) and presence of extract (**b**).

SEM analysis was achieved on the steel surface to define whether the application of a certain dose of the extract under test affected the morphology of the surface. After immersing C-steel in 0.5 M H_2_SO_4_ for 24 h, both with and without the use of 400 ppm of CCE, the C-steel is examined. When C-steel was immersed in 0.5 M H_2_SO_4_ in absence of CCE (Blank), corrosion destroyed the C-steel. After applying the extract, the C-steel surface was examined, and it revealed a smooth, pit-free surface (Fig. 10). The SEM results suggest that the addition of extract leads to the formation of a protective film on the C-steel surface, thereby mitigating corrosion.

#### EDX studies

**Fig. 11:**
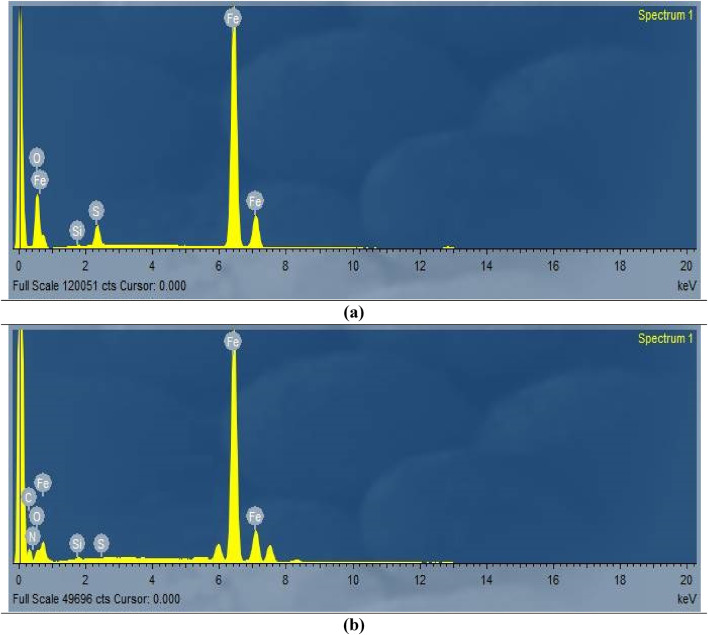
EDX for C-steel surface dipping in 0.5 M sulfuric acid solution for 24 h in the absence (**a**) and presence of extract (**b**).

To support the proposed adsorption mechanism, EDX analysis was performed on carbon steel specimens. Fig. 11 shows EDX spectra of C-steel samples before and after 24-hour immersion in 0.5 M H_2_SO_4_, with and without 400 ppm extract. Table 6 details the elemental composition. Pure C-steel shows Fe and O due to natural oxidation. Inhibitor-free samples show increased O and decreased Fe, indicating the formation of iron oxide. extract-treated samples show increased Fe and additional S and N peaks, suggesting strong adsorption of the extract and the formation of a protective layer.

**Table 6:** EDX data gotten form C-steel surface morphology.

Samples	Wt. %					
	Fe	O	Si	*N*	S	C
Blank	89.4	3.08	0.18	–	3.34	4.0
CCE	72.5	9.37	0.18	10.15	0.01	7.63

#### AFM analysis

**Fig. 12:**
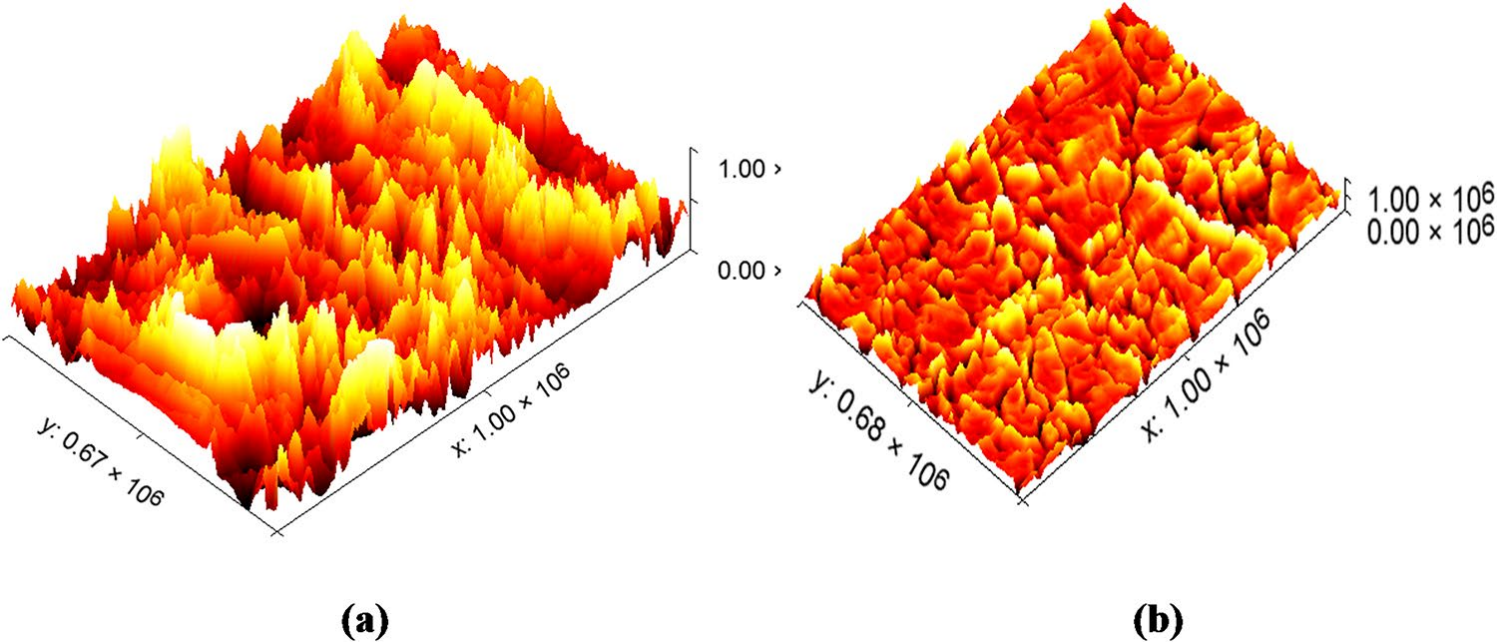
3D AFM pictures (**a**) CS metal earlier dipping in sulfuric acid (blank) and (**b**) presence of 400 for 24 h at 25 ^0^C.

To further investigate the impact of the inhibitor on the metal surface, AFM analysis was conducted on C-steel samples after immersion in 0.5 M sulfuric acid for 24 h, both in the absence and presence of 400 ppm of CCE. Figure 12a illustrates the significant surface damage inflicted by sulfuric acid on the metal. In contrast, Fig. 12b displays the surface morphology of the metal in the presence of 400 ppm of CCE, which remains relatively unaffected by corrosion. The average roughness (R_a_) provides a quantitative measure of surface roughness. The results clearly indicate that the surface of the metal immersed in sulfuric acid is severely damaged, leading to a substantial increase in roughness (546 nm). Conversely, the C-steel sample treated with 400 ppm CCE exhibits a smoother surface with a significantly reduced roughness value (152 nm) compared to the untreated sample. This reduction in surface roughness is a strong indication of the effective adsorption of CCE onto the C-steel surface, resulting in the formation of a protective - that mitigates corrosion.

#### Fourier transform infrared spectroscopy (FT-IR) analysis

**Fig. 13:**
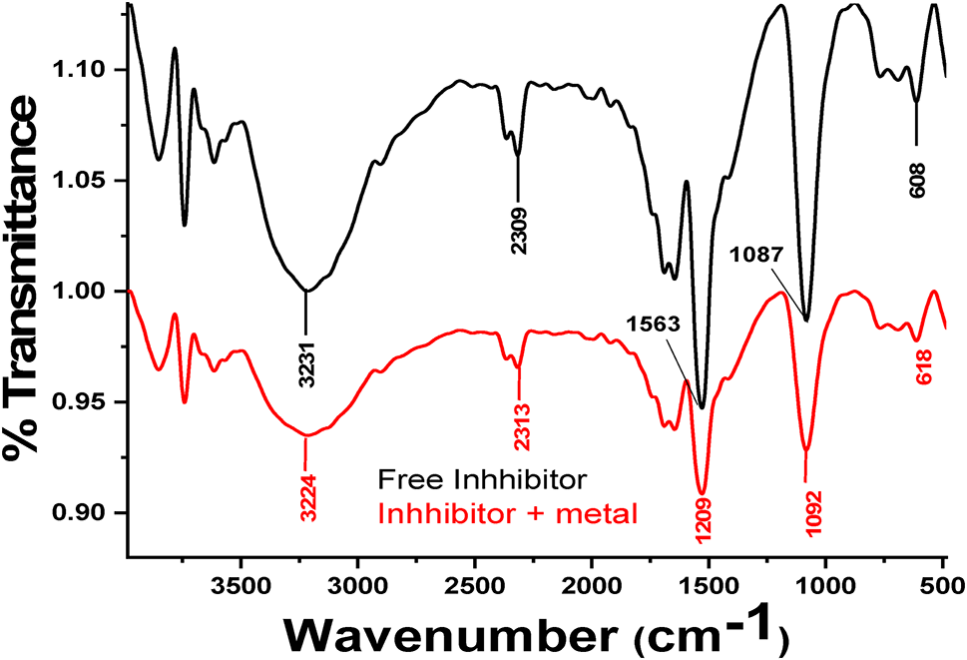
FTIR spectra of *Cuminum cyminum* stock solution (black spectrum line) and adsorbed layer of *Cuminum cyminum* on C-steel surface (the red spectrum line).

The FT-IR spectrophotometer is an effective instrument employed for recognizing the function groups that present in the *Cuminum cyminum* and the type of interaction that occur between function group and metal surface^56,57^. Figure 13 displays broad peaks of *Cuminum cyminum* and *Cuminum cyminum* with C-steel. It is clear that there is some peaks displacement between the spectra of the *Cuminum cyminum* and the adsorbed extract from C-steel surface after corrosion, also a few peaks are disappear or be with less eminent. This indicates the interaction of *Cuminum cyminum* with C-steel through the functional groups presents in *Cuminum cyminum* molecules, resulting in the protection from corrosion occurred.

#### Mechanism of Inhibition

Due to the inherent complexity of adsorption and inhibition processes for any particular inhibitor, it is untenable to posit a singular adsorption mode between the inhibitor and the metal surface. The adsorption of the primary components within the used extract can be ascribed to the existence of oxygen atoms, π-electrons, and aromatic or heterocyclic rings. Furthermore, the presence of a methoxy group serves to augment the inhibition efficacy. Consequently, the potential reaction sites encompass the lone electron pairs of heteroatoms and the π-electrons associated with aromatic or heterocyclic rings. In aqueous acidic environments, the principal constituents are present either as neutral molecules or as protonated molecules, which are cations. Inhibitors can potentially adsorb onto the metal/acid solution interface through one or multiple mechanisms, including: (i) electrostatic interactions between protonated molecules and pre-adsorbed chloride ions, (ii) donor-acceptor interactions occurring between the π-electrons of aromatic rings and the vacant d orbitals of surface iron atoms, and (iii) interactions between the unshared electron pairs of heteroatoms and the vacant d orbitals of iron surface atoms. Typically, two adsorption modes are considered on the metal surface within acidic media. One mode involves the adsorption of neutral molecules onto the C-steel surface via a chemisorption mechanism, which entails the displacement of water molecules from the C-steel surface and the sharing of electrons between heteroatoms and iron. Furthermore, inhibitor molecules are capable of adsorbing onto the C-steel surface through donor-acceptor interactions, specifically between the π-electrons of aromatic or heterocyclic rings and the vacant d-orbitals of surface iron atoms. In the second mode, it is established that the steel surface carries a positive charge within an acidic solution^58^. Consequently, the protonated molecules encounter difficulty in approaching the positively charged C-steel surface (the H_3_O^+^/metal interface) as a result of electrostatic repulsion. Given that SO_4_^2−^ ions are able to introduce an excess of negative charge in the area surrounding the interface, thereby promoting increased adsorption of positively charged inhibitor molecules. Consequently, the protonated inhibitors adsorb through electrostatic interactions between these positively charged molecules and the negatively charged metal surface. This results in a synergistic effect between adsorbed sulfate ions (SO_4_^2−^) and protonated inhibitors. Therefore, the inhibition of C-steel corrosion in 0.5 M H_2_SO_4_ arises from the adsorption of extract constituents onto the C-steel surface. This hypothesis can be further validated through reflectance FTIR analysis of the C-steel surface.

## Conclusions

Based on the findings of this study, we draw the conclusion that the CCE have good inhibitory qualities for API 5 L X70 C-steel in 0.5 molar sulfuric. “The values of inhibition efficiency rise as the dose of the extract does 91.83% at 400 ppm as the maximum at 298 K. PDP studies suggest that the extract functions as a mixed-type inhibitor, affecting both the anodic and cathodic reactions. Impedance data further supports this, indicating that the inhibitory effect is achieved through the adsorption of extract species onto the C-steel surface. The extract under investigation is physically adsorbed across the steel surface, as indicated by adsorption and thermodynamic characteristics, and they conform to the Langmuir adsorption isotherm under all of the studied temperatures. The successful adsorption of the examined extract over the steel surface is confirmed by SEM, EDX and AFM analyses”. SEM analysis reveals the formation of a smooth surface on C-steel in the presence of extract component, likely due to the formation of an adsorptive film of electrostatic character.

## Electronic supplementary material

Below is the link to the electronic supplementary material.

Supplementary Material 1

## Data Availability

The data that support the findings of this study are available from the corresponding author upon reasonable request.
