# Supplementary material for: Cuminum cyminum as green corrosion inhibitor for API 5 L X70 carbon steel in 0.5 M H2SO4 solution
Source: Sci Rep. 2025 May 17;15:17120. doi: 10.1038/s41598-025-98407-z (PMC12084307; doi:10.1038/s41598-025-98407-z)
Supplement: Supplementary file 1 — Supplementary Material 1 [file 41598_2025_98407_MOESM1_ESM.docx]

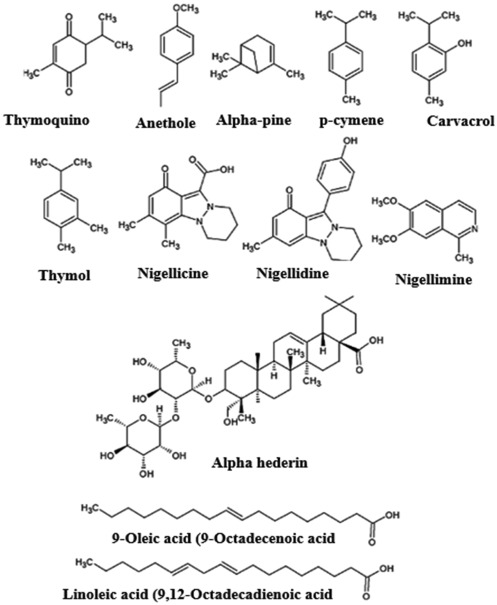


**Fig. S1 Main constituents in Cumin Seed Extract**

**Obtained from** The paper:

Chemical composition, biological activities, uses, nutritional and mineral contents of cumin (*Cuminum cyminum*), **Measurement Food 14 (2024) 100157.**
